# The Equine Gastrointestinal Microbiome: Impacts of Age and Obesity

**DOI:** 10.3389/fmicb.2018.03017

**Published:** 2018-12-07

**Authors:** Philippa K. Morrison, Charles J. Newbold, Eleanor Jones, Hilary J. Worgan, Dai H. Grove-White, Alexandra H. Dugdale, Clare Barfoot, Patricia A. Harris, Caroline McG Argo

**Affiliations:** ^1^Scotland’s Rural College, Aberdeen, United Kingdom; ^2^Scotland’s Rural College, Edinburgh, United Kingdom; ^3^Institute of Biological, Environmental and Rural Sciences, Aberystwyth University, Aberystwyth, United Kingdom; ^4^Faculty of Health and Life Sciences, University of Liverpool, Wirral, United Kingdom; ^5^ChesterGates Veterinary Specialists CVS (UK) Ltd., Chester, United Kingdom; ^6^MARS Horsecare UK Ltd., Buckinghamshire, United Kingdom; ^7^Equine Studies Group, WALTHAM Centre for Pet Nutrition, Leicestershire, United Kingdom

**Keywords:** equine, obesity, age, insulin dysregulation, fecal microbiome, fecal metabolome, apparent digestibility, biomarkers

## Abstract

Gastrointestinal microbial communities are increasingly being implicated in host susceptibilities to nutritional/metabolic diseases; such conditions are more prevalent in obese and/or older horses. This controlled study evaluated associations between host-phenotype and the fecal microbiome / metabolome. Thirty-five, Welsh Mountain pony mares were studied across 2 years (Controls, *n* = 6/year, 5–15 years, Body Condition Score (BCS) 4.5–6/9; Obese, *n* = 6/year, 5–15 years, BCS > 7/9; Aged, *n* = 6 Year 1; *n* = 5 Year 2, ≥19 years old). Animals were individually fed the same hay to maintenance (2% body mass as daily dry matter intake) for 2 (aged / obese) or 4 (control), 4-week periods in a randomized study. Outset phenotype was determined (body fat%, markers of insulin sensitivity). Feces were sampled on the final 3 days of hay feeding-periods and communities determined using Next Generation Sequencing of amplified V1–V2 hypervariable regions of bacterial 16S rRNA. Copy numbers for fecal bacteria, protozoa and fungi were similar across groups, whilst bacterial diversity was increased in the obese group. Dominant bacterial phyla in all groups were *Bacteroidetes > Firmicutes > Fibrobacter*. Significant differences in the bacterial communities of feces were detected between host-phenotype groups. Relative to controls, abundances of *Proteobacteria* were increased for aged animals and *Bacteroidetes*, *Firmicutes*, and *Actinobacteria* were increased for obese animals. Over 500 bacterial operational taxonomic units (OTUs) differed significantly between host-phenotype groups. No consistent pattern of changes in discriminant OTUs between groups were maintained across groups and between years. The core bacterial populations contained 21 OTUs, 6.7% of recovered sequences. Distance-based Redundancy Analyses separated fecal bacterial communities with respect to markers of obesity and insulin dysregulation, as opposed to age. Host-phenotype had no impact on the apparent digestibility of dietary GE or DM, fecal volatile fatty acid concentrations or the fecal metabolome (FT-IR). The current study demonstrates that host-phenotype has major effects on equine fecal microbial population structure. Changes were predominantly associated with the obese state, confirming an obesity-associated impact in the absence of nutritional differences. Clear biomarkers of animal-phenotype were not identified within either the fecal microbiome or metabolome, suggesting functional redundancy within the gut microbiome and/or metabolome.

## Introduction

The gastrointestinal microbiota encompasses the diverse community of microorganisms inhabiting the digestive tracts of mammals. For herbivores, these communities specialize in the fermentation of dietary fiber, providing the body with energy substrates in the form of short-chain fatty acids including acetate, propionate and butyrate. Horses are caeco-colic fermenters, highly adapted to the ingestion (trickle feeding) of plant-based fiber diets. Despite dietary and digestive differences, the microbial community of the equine gut has some similarities to that of humans and is dominated by bacteria from the phyla *Firmicutes* and *Bacteroidetes* (Dougal et al., 2013).

The gut microbiome of the adult human is relatively stable across time, with greater variation between as opposed to within individuals for whom temporal variation is minimal (Costello et al., 2009). Similarly, 65% of the bacterial community within the hindgut of individual ponies was retained across a 6-week period when daily dietary provision was constant (Dougal et al., 2017). Although the composition and structure of the gut microbiome is influenced by host genotype, diet is recognized as the major modifying factor for the human gut microbial community (Wu et al., 2011; David et al., 2014). A typical ‘Western’ human diet rich in protein and fat was associated with a greater number of *Bacteroidetes*. Conversely, diets rich in fiber and carbohydrate were associated with increased proportions of *Prevotella* (Wu et al., 2011). As for man, dietary provision significantly alters the composition of the equine gut microbiome. Transition from forages to feedstuffs rich in rapidly fermentable starch/sugar is associated with rapid and significant changes in the bacterial community composition in fecal samples (Willing et al., 2009; van den Berg et al., 2013; Dougal et al., 2014). Specifically, the incorporation of dietary starch in the form of barley resulted in a decrease in fecal pH, increased lactate concentrations and an increase in the abundance of lactate-producing, *Streptococcus* spp. (De Fombelle et al., 2001).

Furthermore, gut microbiome composition could be expected to influence the efficiency of energy extraction from the diet and thus the availability of excess energy for triglyceride synthesis, and ultimately the development obesity. In this context, the gut microbiome of genetically obese mice has an increased capacity to extract dietary energy (Turnbaugh et al., 2006). Human obesity has been associated with reduced proportions of *Bacteroidetes*, and correspondingly increased abundances of *Firmicutes* in fecal samples (Ley et al., 2006), although this finding has not been consistently replicated (Duncan et al., 2008; Schwiertz et al., 2010). The profound impact of gut microbiome composition on host metabolism has been demonstrated in studies involving the transfer of microbiota from human twins discordant for obesity to germ-free mice resulted in significantly greater body fat gains in those mice receiving microbiota from the obese subject, compared to those receiving microbiota from the lean subject (Ridaura et al., 2013).

Understanding the causal pathways which underpin associations between an obesogenic environment, gut microbiome and adiposity is complex. Numerous studies have identified differences in the composition of the gut microbiome between lean and obese subjects, but many have not been tightly controlled with respect to diet, the primary modifier. A longitudinal murine study recently addressed this deficit by demonstrating that long-term high-fat feeding, as opposed to the obese state *per se*, was the primary driver of changes in the gut microbiome (Kiilerich et al., 2016). This finding was later confirmed using mouse strains with differing susceptibilities to obesity to corroborate that high-fat feeding, rather than the obese state alone was predominant in driving ‘obesity-related’ changes in the gut microbiota (Xiao et al., 2017).

Human obesity is associated with a reduction in insulin sensitivity (Kahn and Flier, 2000), and in turn, a reduced insulin sensitivity has been associated with decreased microbial richness (Le Chatelier et al., 2013). Although a recent study identified some differences in fecal microbiome composition between horses diagnosed with equine metabolic syndrome (EMS: Obese, insulin dysregulated and susceptible to laminitis) and healthy controls (Elzinga et al., 2016), it is unknown whether these differences persist when lean and obese horses are maintained on the same diet.

Age has also been associated with changes in the human gut microbiome. In man, increased age has been associated with a reduction in bacterial diversity and decreases in total *Bacteroidetes* (Woodmansey et al., 2004). Age-related decreases in the ratio of *Firmicutes* to *Bacteroidetes* (Mariat et al., 2009) and changes in the abundance of specific bacterial groups including *Bifidobacterium* and *Enterobacteria* have also been identified (Gavini et al., 2001). Inter-individual variation in the fecal bacterial composition from the human gut has additionally been shown to be greater in elderly subjects compared with younger subjects (Claesson et al., 2011). Although age may be a risk factor for metabolic disease in horses, only one study has identified that aged horses show reduced gut bacterial diversities (Dougal et al., 2014).

The primary objective of this study was to evaluate whether differences in the fecal microbiome of individual animals are associated with host phenotype (largely, age, adiposity, insulin dysregulation, fecal metabolome) and to identify and test any unique operational taxonomic units (OTUs) which could provide robust phenotypic markers. Quality data collection requires a tightly controlled study design. Fecal samples central to this study were collected from 35 rigorously phenotyped, obese, aged and lean ponies receiving the same hay diet. The impacts of confounding factors, including genotype, gender and geographical location were minimized by using a relatively homogenous test population (same breed and gender) maintained under the same, tightly controlled management system throughout.

## Materials and Methods

### Animals and Husbandry

Thirty-five Welsh Section A pony mares were obtained locally (30-mile radius from the University of Liverpool’s Ness Heath Farm) and studied at the same facility over the same 30-week period (May to December) in two consecutive years (Year 1 [2015] *n* = 18; *n* Year 2 [2016] *n* = 17). Recruited animals met selection criteria (below) and were clinically evaluated to be in good general and oral health. Animals had not received any antibiotic treatment in the previous 60 days prior to the commencement of, or at any time during the study. Basal adrenocorticotropic hormone (ACTH) concentrations were measured as a biomarker for pituitary *pars intermedia* dysfunction in plasma samples (Siemens Immulite 1000R, Chemiluminescent Assay) obtained prior to the commencement of the study. Data for all animals were within the normal seasonal range (McGowan et al., 2012). All animals were given appropriate anthelmintic treatment prior to the commencement of the study and routine foot care, vaccination and anthelmintic protocols were maintained throughout the study. Animals were assigned to one of 3 groups (Control, Obese or Aged) on the basis of age and body condition score [BCS, 1 = emaciated to 9 = obese, (Kohnke, 1992) modification of the (Henneke et al., 1983) system] at the time of recruitment (Table 1). Body condition scoring is a widely used method for estimating body fatness in horses and ponies which combines visual appraisal and palpation of subcutaneous body fat across six body regions, scoring each region separately before an overall average is calculated. Selection criteria were as follows: Control animals (*n* = 6/year) were aged between 5 and 15 years with a BCS between 4.5 and 6/9. Obese animals (*n* = 6/year) were selected based on their obese body condition (BCS > 7/9) and were aged between 5 and 15 years. Aged animals (*n* = 6 Year 1; *n* = 5 Year 2) were ≥19 years old.

All procedures were conducted in accordance with Home Office (ASPA) requirements and approved by the University of Liverpool’s Animal Welfare Committee (Home Office project license number: PPL 70/8475). Written informed consent was obtained from all owners.

**Table 1:** Outset phenotype data (mean ± SD) for animals in the current study.

	Aged *n* = 11	Control *n* = 12	Obese *n* = 12
Age (years)	21.55 ± 2.94^b^	9.83 ± 3.21^a^	10.08 ± 3.29^a^
BM (kg)	254.95 ± 31.21^ac^	225.42 ± 18.10^a^	293.83 ± 38.16^b^
Height (cm)	117.64 ± 2.64^a^	117.53 ± 3.67^a^	118.93 ± 3.98^a^
BCS (/9)	6.06 ± 1.24^c^	4.51 ± 0.58^a^	7.98 ± 0.50^b^
Body fat (%)	15.69 ± 7.25^ab^	9.59 ± 6.65^a^	20.75 ± 4.57^b^

*Outset data (mean ± SD) are presented for animal age (years), body mass (BM, kg), withers height (cm), body condition score (BCS, 9-point scale) and body fat percentage (as calculated following deuterium oxide dilution). Different superscripts within rows indicate significant differences between groups.*

### Study Design

The two 30-week study periods were divided into six, consecutive 5-week periods, during which time the animals were fed grass hay from the same batch (over the 2 years) for 4 weeks, at a level predicted to approximate maintenance [2% body mass (BM) as dry matter (DM) intake daily]. Additionally, a proprietary vitamin and mineral balancer (Spillers’ Lite, Spillers, United Kingdom) was fed daily to 0.1% BM. Three replicate samples of each feedstuff were collected and independently analyzed in triplicate. Mean compositions for key nutrients were: Hay; Gross energy (GE), 18.9 MJ/kg DM, Ash, 4.0%, Crude Protein (CP) 8.1%, Acid detergent fiber (ADF), 41.2%, Neutral detergent fibre (NDF) 64.7%, starch. 0.6%, water soluble carbohydrates (WSC) 15.6%. Vitamin / mineral balancer; DE, 9.5 MJ/kg DM, Ash, 14.0%, CP 21.1%, ADF, 14.3%, NDF, 31.7%, starch, 10.5, WSC 12.5%. Daily hay allowances were equally divided and offered as two meals (08.30 and 16.30 h). Nutrient balancer was fed dampened (08.30 h). Individual animal feed provisions were scaled to their entry BM and held constant throughout that period. In any given period within the study, 12/18 animals were ‘on study’ during which animals were individually housed in loose boxes (3 m × 5 m), bedded on wood shavings and had free access to fresh water at all times. Where possible, animals were allowed pasture access for 30 min daily to permit basal exercise and social contact. Grazing was prevented by the use of sealed muzzles (Shires, United Kingdom). When ‘off study’ animals were held at pasture.

Study order was randomized across time to account for any confounding by season. Control animals completed four, 4-week hay-feeding periods, whilst animals in the obese or aged groups underwent two, 4-week hay-feeding periods across the two 30-week study periods.

### Physical Measurements

The BCS, measures of heart and belly girth and BM of all ponies were recorded weekly [BM, nearest 500g, Lightweight Intermediate; Horseweigh, United Kingdom; BCS (Kohnke, 1992)]. BCS and circumferential measures were performed by the same observer on each occasion.

### Fecal Collection

Fresh fecal samples were collected daily for three consecutive days from each individual animal during the final week of hay feeding in each period (days 25–27, inclusive).

The first defecation spontaneously voided by each animal (after 09.00) was immediately sampled with a gloved hand into a clean steel bowl to minimize environmental contamination. On collection, samples were hand-mixed and aliquoted into four, 5 ml sterile vials (Scientific Laboratory Supplies, United Kingdom). Vials were snap-frozen in liquid nitrogen within 5 min of collection and stored at −80°C prior to DNA extraction.

### Estimates of Total Body Composition

Total body water (TBW) and total body fat mass were calculated for each individual animal during the final week of the first hay feeding period for that animal using the deuterium oxide (D_2_O) dilution method, as previously described and validated for clinical use in the pony (Dugdale et al., 2011b). Deuterium enrichments in plasma samples were analyzed in duplicate by a commercial laboratory (Iso-analytical, Cheshire, United Kingdom) by gas isotope ratio mass spectrometry.

### Combined Glucose-Insulin Tolerance Test (CGIT)

To generate individual animal indices of insulin sensitivity, a dynamic CGIT was performed on all animals during the final week of the first hay feeding period (Argo et al., 2012). Blood samples were immediately transferred to lithium heparinized tubes (BD vacutainer), mixed and placed on ice prior to centrifugation (2000 g for 10 min). Plasma was aliquoted in duplicate and stored at −20°C prior to analysis. Plasma glucose samples were analyzed using the hexokinase method. Plasma insulin concentrations were measured using a chemiluminescent assay (Siemens Immulite 1000R, Chemiluminescent Assay).

### Apparent Digestibility

Total fecal collections were conducted for individual ponies over 3 consecutive days (72 h) during the final week of each hay feeding period. Any refused feed was recorded at the end of each 24-h period. Total daily fecal collections were weighed, thoroughly mixed and duplicate samples were collected for analysis. Dry matter (DM) contents of feces were determined by drying (70°C) duplicate samples (∼250 g) to constant mass. Ash contents were recorded following combustion of duplicate DM samples at 550°C (Carbolite OAF:1; Carbolite Furnaces). The three dry fecal samples collected over a 72-h period were ground, pooled and homogenized for the evaluation of GE content (MJ/kgDM) by bomb calorimetry at a commercial laboratory (Sciantec, United Kingdom).

### DNA Extraction and Quantitative PCR

Genomic DNA was extracted from freeze-dried fecal samples (25 mg DM) which were bead-beaten in 4% SDS lysis buffer for 45 s. DNA was extracted using a CTAB/Chloroform method (adapted from Yu and Morrison, 2004) in that lysis of cells was achieved by incubating with sodium dodecyl sulfate (SDS) buffer for 10 min at 95°C and potassium acetate was substituted for phenol in removing proteins. Concentrations and qualities of genomic DNA were assessed by spectrophotometry (Nanodrop ND-100, Thermo Scientific, United States). Absolute concentrations of DNA from total bacteria, protozoa and fungi were determined by qPCR and serial dilutions of their respective standards (10^1^ to 10^5^) as previously described (Belanche et al., 2012, 2016). Quantitative PCR (qPCR) was conducted in triplicate using a LightCycler 480 System (Roche, Mannheim, Germany).

### Ion Torrent Next Generation Sequencing

Bacterial communities were studied using Next Generation Sequencing (NGS) (de la Fuente et al., 2014). For bacterial profiling, amplification of the V1–V2 hypervariable regions of the 16S rRNA was carried out using bacterial primers (27F and 357R) followed by Ion Torrent adaptors. Forward primers were barcoded with 10 nucleotides to allow sample identification. PCR was carried out in a 25 μL reaction vessel containing DNA template (1 μL), 0.2 μL reverse primer, 1 μL forward primer, 5 μL buffer (PCR Biosystems Ltd., London, United Kingdom), 0.25 μL bio HiFi polymerase (PCR Biosystems) and 17.6 μL molecular grade water. Amplification conditions were 95°C for 1 min, then 22 cycles of 95°C for 15 s, 55°C for 15 s and 72°C for 30 s. To assess the quality of amplifications, resultant amplicons were visualized on a 1% agarose gel. PCR products were then purified using Agencourt AMpure XP beads (Beckman Coulter Inc., Fullerton, United States) and DNA concentration was determined using an Epoch Microplate Spectrophotometer fitted with a Take 3 Micro-Volume plate (BioTek, Potton, United Kingdom) to enable equimolar pooling of samples with unique barcodes.

Libraries were further purified using the EGel system with 2% agarose gel (Life Technologies Ltd., Paisley, United Kingdom). Purified libraries were assessed for quality and quantified on an Agilent 2100 Bioanalyzer with High Sensitivity DNA chip (Agilent Technologies Ltd., Stockport, United Kingdom). Library preparation for NGS sequencing was carried out using the Ion Chef system (Life Technologies UK Ltd.) and the Ion PGM HiQ Chef Kit, and sequencing using the Ion Torrent Personal Genome Machine (PGM) system on an Ion PGM Sequencing 316 Chips v2 BC.

Following sequencing, data were processed as previously described (de la Fuente et al., 2014). Briefly, sample identification numbers were assigned to multiplexed reads using the MOTHUR software environment (Schloss et al., 2009). Data were de-noised by removing low-quality sequences, sequencing errors and chimeras (quality parameters: maximum 10 homopolymers, qaverage 13, qwindow 25, for archaea the qwindow was set at 30, and erate = 1; Chimera check, both *de novo* and database driven using Uchime). Sequences were clustered into OTUSs using the Uparse pipeline at 97% identity. Bacterial taxonomic information on 16S rRNA sequences was obtained by comparing against Ribosomal Database Project-II (Wang et al., 2007). The number of reads per sample were normalized to the sample with the lowest number of sequences.

### Measurement of Fecal Volatile Fatty Acid Concentrations

Fecal samples were defrosted and diluted 1:5 w/v with distilled H_2_O (2 g sample/8 mL H_2_O) to make a fecal slurry and pH was recorded prior to the addition of 20% orthophosphoric acid (containing 20 mM 2-ethyl butyric acid as an internal standard) also at 1:5 (1 mL acid/4 mL fecal slurry) to deproteinise the samples. For VFA analyses, slurries were left for 24 h to allow sediments to settle before being syringe-filtered through a glass-fiber pre-filter (0.7 μm pore, Millipore) and a nitrocellulose membrane (0.45 μm pore; Millipore) into a glass vial and capped. VFAs were determined by gas liquid chromatography using ethyl butyric acid as the internal standard as described by Stewart and Duncan (1985).

### Characterization of the Fecal Metabolome: FT-IR Spectroscopy (Reflectance Mode)

Samples were defrosted, diluted with distilled water (1:5), vortexed briefly and 20 μL were added onto a 100-well FT-IR plate. Plates were dried overnight at 50 °C before analysis; for each sample, three machine replicates were prepared to account for variation. Samples were analyzed by FT-IR using a Vertex 70 spectrophotometer (Bruker Optik GmbH, Germany). Triplicate infrared spectra were collected and read at a range of 4000–600 cm^−1^ and a resolution of 4 cm^−1^. The software used for data collection was OPUS (version 6.5; Bruker Optik GmbH).

### Statistical Analyses

Phenotypic data (BM, morphometric data, CGIT data, etc.) were entered into Excel then exported for analysis into STATA 13 (StataCorp, TX, United States). To investigate changes in BM during hay feeding periods, univariate mixed regression models with pony ID as a random effect were fitted with the logit transformed proportional BM (adjusted to Week 1 during each hay feeding period to account for changes in gut-fill during the 1st week on hay) as the outcome variable. Explanatory variables were previous diet, study period and group. Variables with a *P*-value ≤ 0.15 were offered to a multivariable model fitted using a backward stepwise elimination strategy. To evaluate group differences in outset CGIT parameters, one-way ANOVA was employed. Univariate regression models were fitted to evaluate associations between measures of microbial diversity and host-phenotype (e.g., CGIT parameters, body fat percentage, digestibility), with diversity measures being the outcome variables and phenotypic measures as explanatory variables.

### Microbiome Analysis

Simpson and Shannon–Wiener diversity indices were calculated using normalized data as recommended to reduce over-inflation of true diversity in pyrosequencing data sets (Gihring et al., 2012). Species richness and diversity were then analyzed by two-way ANOVA using GenStat^®^ 15th edition (VSN International, Hemel Hempstead, United Kingdom), unless otherwise mentioned below. *P*-values were considered significant if < 0.05.

Phylum and Genera level differences in the microbiome with respect to animal group and study period were investigated by ANOVA. Statistical analyses excluded those for which an abundance of less than 0.05% was recorded. *P*-values were adjusted for multiple testing using the method proposed by Benjamini and Hochberg (Benjamini and Hochberg, 1995) to decrease the false discovery rate. When effects were detected, treatment means were compared by Fisher’s protected least significant difference (LSD) test. Findings with *P* < 0.10 when applying Benjamini and Hochberg (1995) correction were regarded as statistically significant. Genstat 15th Edition (VSN International, Hemel Hempstead, United Kingdom) was used.

Differential abundances at an OTU level were evaluated using the bioconductor package DESEQ21 in the statistical package R, a methodology appropriate for the interrogation of high-throughput, sequencing count data, allowing models to be built using a negative binomial distribution to account for the distribution of read counts from each OTU (Love et al., 2014).

Permutation multivariate analysis of variance (PERMANOVA) was used to determine overall significant differences in bacterial and archaea communities. Analyses were performed in PRIMER 6 & PERMANOVA+ (versions 6.1.18 and 1.0.8, respectively; Primer-E, Ivybridge, United Kingdom). Abundance percentage data were subjected to square-root transformation and Bray–Curtis distance matrices were calculated. PERMANOVA was carried out using default settings with 9999 unrestricted permutations and the Monte Carlo *P*-value was calculated. Analyses of Similarity (ANOSIM) were conducted in PRIMER 6 & PERMANOVA+, using the Bray–Curtis distance matrix calculated above. This analysis was used to provide a metric of the degree of divergence between communities, as given by the R statistic.

Fecal VFA concentrations were analyzed by ANOVA (Genstat^®^ 12th edition; VSN International Ltd.) and comparisons between phenotypic groups were conducted by using Fisher’s LSD test. Statistical significance was considered if *P* < 0.05.

FT-IR data were analyzed according to the method described by Elliott et al. (2007), using discriminant function analysis (DFA), optimized by cross-validation. An optimal configuration was found using varying numbers of principal components to account for the majority of variance within the samples. In this instance, one-way ANOVA was used to establish a significant difference between groups (*P* < 0.05). This was combined with Tukey’s honestly significant difference (HSD) test to make multiple comparisons in a pairwise method to ascertain where exactly, differences existed. All data were analyzed using the Matlab^®^ interactive environment [Version 7.8.0 (R2009a); MathWorks^®^, Natick, MA, United States] using in-house scripts. Pairwise differences between significantly different wavenumbers from groups were generated according to the study by Enot et al. (2008).

To calculate the contribution of environmental data on bacterial communities, distance-based linear modeling was used to calculate which environmental variables had a significant correlation with the community data. Significant variables were used in distance-based redundancy analysis (dbRDA) (Legendre and Anderson, 1999) as implemented in PRIMER 6 and PERMANOVA+.

### Nucleotide Sequence Accession Numbers

Raw sequences reads from the bacterial libraries were deposited at the EBI Short Read Archive from of the European Nucleotide Archive (accession number PRJEB29667).

**Table 2:** Combined glucose-insulin tolerance test and apparent digestibility parameters (mean ± SD) for animals in the three phenotypic groups.

	Aged (*n* = 11)	Control (*n* = 12)	Obese (*n* = 12)
Baseline insulin (μIU/ml)	8.17 ± 8.22^b^	2.13 ± 0.42^a^	6.93 ± 5.27^ab^
Insulin time 45 (μIU/ml)	114.70 ± 79.27^ab^	47.6 ± 29.92^a^	116.15 ± 95.79^b^
Insulin time 75 (μIU/ml)	53.86 ± 58.99^ab^	12.35 ± 7.17^a^	86.89 ± 84.27^b^
AUC insulin (μIU/ml/min)	5292.86 ± 3849.65^ab^	2018.03 ± 1231.08^a^	7689.0 ± 4911.28^b^
Baseline glucose (mmol/L)	5.48 ± 0.59^a^	5.23 ± 0.57^a^	5.33 ± 0.51^a^
AUC glucose (mmol/L/min)	914.18 ± 178.11^a^	774.60 ± 121.13^a^	946.06 ± 147.42^a^
Return to baseline glucose concentration (minutes)	79.09 ± 50.09^a^	50 ± 24.49^a^	83.33 ± 45.14^a^
DM digestibility (%)	51.78 ± 1.92^a^	50.91 ± 1.54^a^	50.27 ± 1.62^a^
GE digestibility (%)	51.41 ± 5.00^a^	49.40 ± 4.28^a^	48.84 ± 3.82^a^

*Data (mean ± SD) are presented for the control (n = 12), obese (n = 12), and aged (n = 11) pony groups. Outcome values for the combined glucose-insulin tolerance test (CGIT) were fasted baseline values for plasma glucose and insulin concentrations, insulin concentrations 45 and 75 min post-infusion, the areas under the curves (AUC) for insulin and glucose and the time taken for glucose concentrations to return to baseline values. The apparent digestibilities of gross energy (GE) and dry matter (DM) are also shown. Different superscripts within rows indicate significant between-group differences.*

## Results

### Body Weight Changes During Hay Feeding Periods

All animals remained healthy throughout. The plane of nutrition (2% of BM as daily hay DMI) was selected to approximate maintenance requirements. Overall, 33/35 (94%) of animals remained within 5% of Week 1 body mass during all hay feeding periods, and of these 33 animals, 18 (55%) remained within 2% of Week 1 body mass during all hay feeding periods. Overall mean percentage change in body mass (±standard deviation; adjusted to Week 1) between groups was as follows: Control: 0.02% (±2.13); Obese: 1.01% (±1.92); Aged: 0.18% (±1.93). Univariate regression analysis revealed no effect of group, study period, or previous diet on proportional BM changes during hay feeding periods, therefore multivariable modeling was not performed. Body condition scores (Kohnke, 1992) remained relatively unchanged within hay feeding periods, with overall mean changes (± standard deviation) during hay feeding periods as follows: Control 0.12 ± 0.27 points; Obese 0.06 ± 0.26 points; Aged −0.17 ± 0.37 points.

### Body Composition and Insulin/Glucose Dynamics

Mean body fat percentage in the obese group (20%) was twice that of control animals (10%) while the aged group were intermediate based on both deuterium oxide dilution and BCS (Table 1). Plasma insulin and glucose concentrations during the dynamic CGIT test, revealed significant differences between phenotypic groups. On the basis of fasted samples, all ponies were normoinsulinaemic and normoglycaemic at outset (Frank et al., 2010). Mean fasted baseline concentrations of plasma insulin in aged ponies, although within the normal range (<20IU/ml), were significantly greater than those of control animals (*p* < 0.05), while values for obese ponies occupied an intermediate range (Table 2). Conversely, mean concentrations for plasma insulin measured at 45 and 75 min post-infusion and the areas under the curves for both insulin (AUCi) and glucose (AUCg) were significantly greater for the obese group compared to the control group (*p* < 0.05, Table 2) while variation among aged animals overlapped these ranges. There were no group differences in the time taken to return to baseline glucose. Overall, there was less variation in CGIT responses for control animals than recorded for animals in the older or obese groups (Table 2). Across all animals, there were some positive associations between markers of insulin dysregulation and outset body fat percentage (AUCi, *R*^2^ = 0.14, *p* = 0.03; iT0, *R*^2^ = 0.17, *p* = 0.02; iT45, *R*^2^ = 0.15, *p* = 0.02; iT75, *R*^2^ = 0.07, *P* = 0.12; AUCg, *R*^2^ = 0.19, *p* = 0.01; Return to baseline glucose concentration (RTBg), *R*^2^ = 0.04, *p* = 0.26, Supplementary Table S1).

### Apparent Digestibility

The apparent digestibility of dry matter (DM) and gross energy (GE) was similar for all three animal groups. Overall, the mean apparent GE digestibility (± SD) was 49.9 ± 1.0% (Table 2). Mean (± SD) digestible energy intakes (DEI) were similar across groups irrespective of how these were calculated; 0.19 MJ/kgBM, 0.64 MJ/kgBM^0.75^, 0.22 MJ/kg lean BM, or 0.86 MJ/kg lean BM^0.75^. There was no effect of study period or year on the apparent digestibility of DM, GE or relative DE intakes.

### Characterization of Fecal Bacteria

There were no significant differences between groups in the copy number of total bacteria, protozoa or fungi as analyzed by q-PCR (Table 3). Quality filtering of 16S rDNA amplicon sequences resulted in 11,089,862 high-quality sequences (320 bp long) which clustered in 9672 different OTUs. A phylogenetic tree was constructed (PRIMER 6 with Bray–Curtis dissimilarity, Supplementary Figure S1), which indicated that samples from an animal, collected on each of the three successive sampling days, tended to cluster together. This observation allowed data arising from these serial samples to be pooled for each animal within a specific study period. Pooled analyses provided 12,000 sequences per animal, per period after normalization. Rarefaction curves (Supplementary Figure S2) demonstrated that sample curves for each phenotypic group had not plateaued; indicating that complete sampling of these environments had not yet been achieved.

**Table 3:** Copy numbers for total bacteria, protozoa and fungi in the feces of the 3 phenotypic groups.

	Aged (*n* = 11)	Control (*n* = 12)	Obese (*n* = 12)
Total bacteria	10.88 ± 0.09	10.79 ± 0.26	10.87 ± 0.14
Protozoa	9.30 ± 0.60	9.10 ± 0.43	9.36 ± 0.46
Fungi	8.39 ± 0.42	8.35 ± 0.53	8.28 ± 0.50

*Total bacteria, protozoa, fungi (log copy/g DM; Mean ± SD).*

There was no difference in either observed species (S.Obs) or in Chao 1 estimates of species richness between phenotypic groups (Table 4). However, the fecal microbiome of obese animals was significantly more diverse in terms of bacterial species when compared to aged and control animals (Shannon index; *p* = 0.02; Table 4). Obese animals also had a significantly greater Simpson index compared to control animals (*p* = 0.02; Table 4), indicating lower evenness in the distribution of bacterial species present. Mean Shannon and Simpson indices for aged animals also demonstrated a tendency to be increased over control animals (Table 4). No associations were identified between diversity indices and outset phenotype measurements (CGIT parameters, body fat percentage, digestibility).

**Table 4:** Diversity and richness indices for fecal bacterial communities across host phenotypic groups.

	Aged (*n* = 11)	Control (*n* = 12)	Obese (*n* = 12)	SED	*P*-value
Simpson’s diversity	0.98^a^	0.96^a^	0.99^b^	0.01	0.02
Shannon–Wiener diversity	6.04^a^	5.76^a^	6.23^b^	0.21	0.02
S.Obs	2371	2320	2578	142.2	0.09
S.Chao1	4278	4113	4713	499.4	0.35

*Mean diversity indices (Simpson’s and Shannon–Weiner), species observed (S.Obs) and estimated species richness (S.Chao1) for fecal samples collected over the final 3 days of hay feeding across host phenotypic groups. Significant differences between groups are denoted by different suffixes.*

**FIGURE 1:**
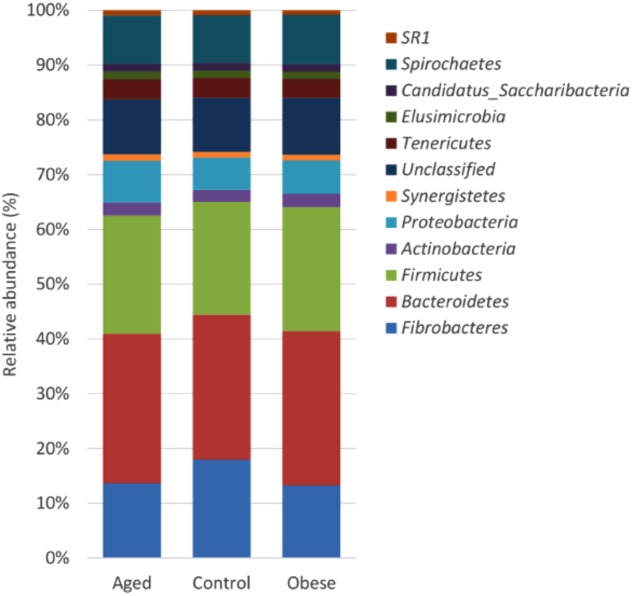
Stacked histogram illustrating the relative abundance of the dominant bacterial phyla (present at > 0.05%) across host phenotypic groups [Aged (*n* = 11), Control (*n* = 12), and Obese (*n* = 12)].

Across all animal phenotypic groups, the most abundant phyla were the *Bacteroidetes*, followed by the *Firmicutes* and *Fibrobacteres* (Figure 1). The relative abundance of *Bacteroidetes*, *Firmicutes* and *Actinobacteria* was significantly greater in obese compared to control animals, whilst the relative abundance of *Proteobacteria* was significantly greater in the aged group compared to the control group. However, the greatest observed phylum-level difference was that *Fibrobacteres* were significantly more abundant in control animals than in either the aged or obese groups (Supplementary Table S2). Neither study period nor year had an impact on the relative abundance of individual phyla.

**FIGURE 2:**
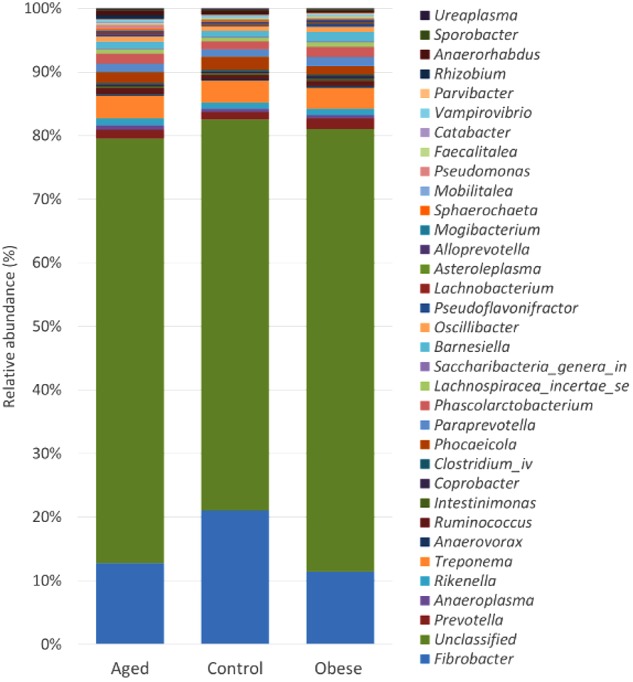
Stacked histogram illustrating the relative abundance of the dominant bacterial genera (present at > 0.05%) across host phenotypic groups [Aged (*n* = 11), Control (*n* = 12), and Obese (*n* = 12)].

Although many OTUs were not conclusively characterized at a genus level, *Fibrobacter* was the most abundant genus in all animals (Figure 2). The abundance of *Fibrobacter* was significantly higher in control compared to both aged and obese animals (Supplementary Table S3). The abundance of *Pseudoflavonifractor* and OTUs unclassified at a genus level was significantly lower in control compared to both aged and obese animals (Supplementary Table S3). No effect of study period or year was observed on the relative abundance of individual genera.

### Bacterial Species Between Groups

At an OTU level, 524 bacterial species differed statistically in abundance between phenotype groups (Supplementary Tables S4–S6). However, when animals were considered by year of study to test for the resilience of these observations as phenotypic markers, of the 37 OTUs which differed between control and obese animals in Year 1, only four remained as significant differences between these groups in animals studied in Year 2 (OTU 87, a *Firmicutes* classified down to the level of *Ruminococcaceae*; OTU 154, a *Bacteroidetes;* OTU 629 a *Firmicutes* classified to the level of *Lachnospiraceae* and OTU 933, a *Bacteroidetes*). However, 2 of these 4 distinctive OTUs differed in their direction of change between study years; i.e., OTU 87 was more abundant in control animals in Year 1 but more abundant in obese animals in Year 2. The opposite was true for OTU 154 (Supplementary Table S7). Similar results were evident with comparisons between control and aged animals and aged and obese animals with no consistent pattern changes in discriminant OTUs between groups being observed in both years (Supplementary Tables S4, S5).

### Microbiome Structure

PERMANOVA analysis revealed significant differences in community data between groups (*p* < 0.05; Table 5), and this was confirmed by ANOSIM analysis. The greatest divergence between groups was observed between control and aged groups (*R* = 0.31), followed by control and obese groups (*R* = 0.11).

**Table 5:** Summary of ANOSIM and PERMANOVA outputs.

	Group	Control	Obese
PERMANOVA (*P*-value)	Obese	0.001	
	Aged	0.001	0.011
ANOSIM (*R*-value)	Obese	**0.11**	
	Aged	**0.31**	**0.07**

*Results matrices for the fecal microbiomes recovered from control, obese and aged horses. Significant P-values for PERMANOVA (p < 0.05) are highlighted. ANOSIM R-values indicate the degree of separation between samples (0 = very similar; 1 = highly dissimilar), with significant R-values (with p < 0.05) shown in bold.*

### Core Bacterial Community

The core bacterial community was defined as bacteria present in all samples analyzed in the current study at a relative abundance of 0.1% or greater. A total of 21 OTUs made up the core bacterial community in the current study, accounting for on average 6.67% total sequences recovered (7.11% aged group; 6.55% control group; 6.37% obese group). The core community was comprised primarily of bacteria belonging to the *Bacteroidetes* phyla, followed by the *Firmicutes* (Supplementary Figure S3). When the relative abundance of the OTU core community was compared between groups, no significant differences were found for any of the 21 core OTUs (Supplementary Table S8).

### Effect of Phenotype on the Fecal Metabolome

**FIGURE 3:**
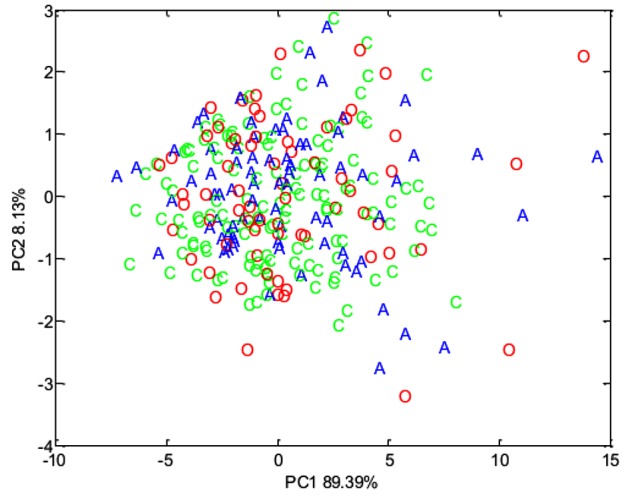
Principal component analysis (PCA) plot summarizing fecal metabolome data derived from the host phenotypic groups: Aged (A) (*n* = 11), Control (C) (*n* = 12), and Obese (O) (*n* = 12).

Concentrations of volatile fatty acids in the feces were similar for all three phenotypic groups, irrespective of whether data were combined across the study (Table 6) or analyzed separately by year. However, fecal pH was lower in control animals, both in the total data set (*p* < 0.01, Table 6). No effect of animal phenotype was evident on the total fecal metabolome determined by FT-IR (Figure 3).

**Table 6:** Mean pH and volatile fatty acid concentration in feces from obese, aged and control horses fed a diet of hay.

	Aged (*n* = 11)	Control (*n* = 12)	Obese (*n* = 12)	SED	*P*-value
pH	6.50	6.35	6.53	0.05	<0.01
Acetate (mM)	23.26	25.7	25.52	1.60	0.12
Propionate (mM)	7.26	7.76	7.40	0.62	0.62
Butyrate (mM)	2.56	2.74	2.64	0.20	0.57
BCVFA (mM)	2.92	3.04	3.16	0.29	0.68

*BCVFA, Branched chain volatile fatty acids.*

### Associations Between Microbiome and Host Phenotype

**FIGURE 4:**
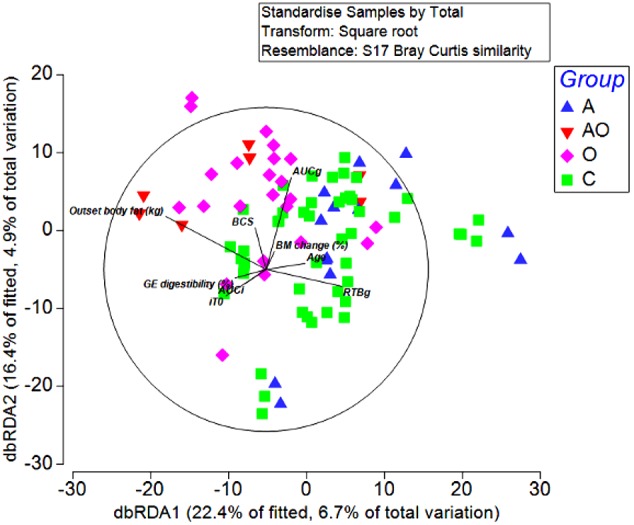
Distance-based redundancy analysis illustrating associations between the fecal microbiome of Aged (A), Aged and Obese (AO), Obese (O), and Control (C) animals and outset host phenotype measures; Apparent digestibility of gross energy (GE digestibility), body condition score (BCS), areas under the curves for insulin (AUCi) and glucose (AUCg), and the time taken for glucose concentrations to return to baseline (RTBg) during a combined glucose/insulin tolerance test, outset body fat (kg), age (years), and percentage changes in body mass across the 4 week hay feeding periods [BM change (%)].

**FIGURE 5:**
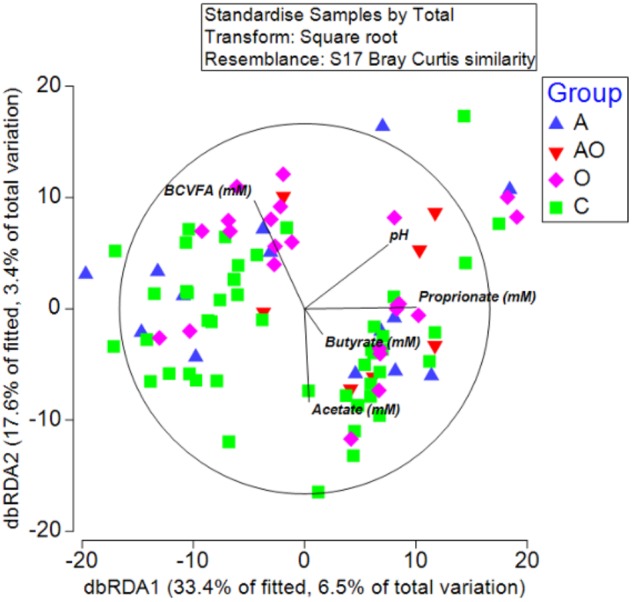
Distance-based redundancy analysis illustrating associations between microbiome and fecal metabolome measures (branched chain volatile fatty acids (BCVFA), concentrations (mM) of acetate, butyrate and propionate and fecal pH) in aged (A), aged and obese (AO), obese (O), and control (C) animals.

To detect possible correlations between the structure of the bacterial community and animal phenotype, a distance-based redundancy analysis (dbRDA) was performed. However, the primary axis accounted for only 22% of the fitted variation. A separation in terms of BCS and total body fat was evident for obese animals and in those aged animals classified as obese (BCS > 7/9), suggesting a difference in the microbiome structure of obese animals (Figure 4). However, whilst attempts to relate population structure to concentrations of volatile fatty acids in feces described 33 % of the fitted variance in the first axis and 17% in the second axis, no clear relationships between fecal volatile fatty acids and the structure of the fecal microbiome were evident (Figure 5).

## Discussion

The ability to exert strict nutritional and environmental control on a significant number of ponies of common breed and gender provided a unique platform to critically evaluate associations between host phenotype and the fecal microbiome / metabolome.

While recruitment criteria for control and obese animals were readily satisfied in terms of age and adiposity, inclusion of obese animals within the healthy-aged group was unavoidable. The spectrum of BCS and total body fat mass recorded for these older animals (Table 1) agreed with published observations of the wider aged equine community (Ireland et al., 2012b). A primary objective of this study was to identify changes associated with altered gastrointestinal function in older animals. Improvements in health care and decreased workloads throughout life have resulted in the majority of leisure animals entering old age in good health and, before the advent of the ultimately unavoidable senescent deterioration, aged ponies are as likely to be over-conditioned as thin (Ireland et al., 2012a). While clear group differences in total body fat and age were evident between the obese and aged groups relative to the controls, older animals were distributed across a much wider range in terms of body fat content and key markers of insulin sensitivity /dysregulation (Funk et al., 2012). On consensus, the aged group were intermediately positioned relative to obese and control animals and data should be interpreted accordingly.

The plane of nutrition selected for maintenance of BM (2% of BM as daily DMI) was appropriate for the majority of animals and within constraints on DM appetite for obese, Welsh Mountain pony mares (Dugdale et al., 2010). That the apparent digestibility of dietary DM and GE was independent of host phenotype was an unexpected finding given the marked differences observed in gut bacterial microflora. Previous studies using the Welsh pony mare model have also failed to detect changes in digestibility between season and following weight loss / gain (Dugdale et al., 2010, 2011a). Despite individual variation in BM change across the hay-fed periods, all animals remained within ±5% of the BM recorded at the end of their 1st week of hay feeding, suggesting a maintenance energy requirement of 0.19 MJ/kg BM^0.75^ / day. This value exceeded estimates of DE requirements for maintenance derived by regression of DEI on ADG for obese pony mares (0.16 MJ / kg BM^0.75^; Dugdale et al., 2011a). The greater energy requirement of animals in the current study may be partially attributed to between-study differences in animal management, exercise provision and the inclusion of metabolically more demanding, older and leaner animals in the current data set. Failure to detect any group differences in weight gain/loss was supported by the lack of impact of either obesity or age on digestibility of dietary energy or DM. It was surprising that period had no effect on weight change or estimates of energy requirements. Previous studies have clearly revealed seasonal changes in DE requirements for energy balance (Fuller et al., 2001; Dugdale et al., 2011a).

This study used equine feces to investigate changes in the gut bacterial population in control, aged and obese ponies fed a consistent diet. Feces are often used for microbial investigations as well as for seeding media to conduct *in vitro* studies of nutrient digestibility. Although the fecal microflora is commonly accepted as a suitable representation of the microbial population of the equine hind-gut, limited attention has been given to evaluating this relationship. Previous studies (Al Jassim and Andrews, 2009; Dougal et al., 2012, 2013; Ericsson et al., 2016) suggest that both the microbiome and associated metabolome of digesta vary across different regions of the equine digestive tract. Data from these studies support that while fecal samples are likely to offer a fair representation of the microbial population of the right dorsal colon, they are less representative of the microflora within the caecum and small intestine. Data from the current study should be interpreted in this context.

In contrast to previous studies in humans where microbial diversity was significantly reduced for obese individuals (Turnbaugh et al., 2009) and our previous observations in ponies, in which weight loss failed to markedly alter fecal bacterial diversity (Dougal et al., 2017), this study clearly identified that the bacterial diversity of feces was significantly increased in obese ponies and tended toward a significant increase in aged ponies. These data are at odds with the findings of Dougal et al. (2014) who reported decreased bacterial species richness and diversity in elderly (8 × 20 and 1 × 28 year) as opposed to younger (5–12 years), healthy stock-horse mares. While the current study used older ponies of diverse BCS, the earlier study was conducted with horses, all of which were in moderate BCS. Study differences include single fecal samplings at the end of 3 week feeding periods using 3 different diets (Dougal et al., 2014) as opposed to the current report of samples collected over 3 consecutive days at the end of 4 weeks of hay feeding alone. Furthermore, differences in longevity between horses and ponies might suggest that the horses were more likely to be experiencing or developing early senescent changes. The onset of senescence may be a likely contributor for decreased diversity within the fecal microbiome, while the chronologically similar but physiologically younger ponies demonstrated relatively increased diversities of fecal bacteria.

The most abundant bacterial phyla within equine feces tend to be *Bacteroidetes* and *Firmicutes*, with smaller quantities of *Spirochaetes, Fibrobacteres, Proteobacteria*, and *Actinobacteria* (Dougal et al., 2013, 2014), an observation consistent with the overall data presented for this study. We have previously observed age- and body mass-related trends in the bacterial community within equine feces (Dougal et al., 2012, 2017), although animal numbers were insufficient to directly relate the fecal microbiome with animal phenotypes. Amongst the major phyla which comprised more than 10% of the total bacterial population in the current study, there were significant effects of host phenotype on *Fibrobacteres, Bacteroidetes* and *Firmicutes.* In agreement with the current findings, a recent study identified an increased bacterial diversity and abundance of *Firmicutes* in obese horses (Biddle et al., 2018). However, obese animals also had reduced proportions of *Bacteroidetes* and *Actinobacteria* in that study (Biddle et al., 2018), whereas the current study identified increased abundance of *Bacteroidetes* and *Actinobacteria* in the obese group. The current study utilized more strictly controlled design in terms of diet and breed-type of animals studied which may be responsible for the differences in the findings. In humans, changes in the abundance of the *Bacteroidetes* and *Firmicutes* phyla have been associated with obesity (Schwiertz et al., 2010; Remely et al., 2013; Koliada et al., 2017). However, this was not the case for the current equine study, and a recent meta-analysis evaluating differences in the fecal microbiome between lean and diet-induced obese rodents identified inconsistencies in the *Bacteroidetes* to *Firmicutes* ratio between studies, and the authors suggested that it is not a useful indicator of an obese phenotype (Jiao et al., 2018).

In this context, it is important to consider differences within microbial genera, as differences at the phylum level may be misleading (Zhang et al., 2009). In the current study, *Pseudoflavonifractor* (belonging to the butyrate-producing *Firmicutes* pylum) was more abundant in obese animals and abundance of this genus has previously been associated with the gut microbiota of obese humans sensitive to a controlled weight loss intervention (Louis et al., 2016). However, the inter-species relevance of this finding remains to be determined. An increased proportion of *Fibrobacter* in the control group compared to obese or aged ponies in the current study might suggest an increased capacity for fiber digestion as bacterial species belonging to the *Fibrobacter* genus are known for their fibrolytic activity (Daly et al., 2012). However, although there were no differences observed between the groups in the fecal concentrations of VFAs, the control group were found to have a lower fecal pH compared to the other groups which may also suggest differences in the production and/or absorption of the individual VFAs across the large intestine into the bloodstream (van den Berg et al., 2013). Further studies are required to ascertain the relationship between bacterial metabolism and impacts on VFA turnover in the horse.

Deseq analysis of the complete data set in the current study suggested that key OTUs might be identified that clearly differentiated between the different phenotypic states. However, attempts to validate specific OTUs as phenotypic markers did not stand up to preliminary scrutiny. Key indicator OTUs identified in half the animals (Year 1 cohort), were unable to independently predict the phenotype of the remainder population (Year 2 animals). Consistent changes in the bacterial structure, predictive of phenotypic status were not recognized. This may be a limitation of the number of animals used (6 per year per phenotype) or possibly it was a consequence of phenotypic plasticity within gut microbial populations, in which the ability of multiple species to fill individual metabolic niches represents a degree of functional redundancy (Lozupone et al., 2012; Moya and Ferrer, 2016).

Over recent years, the concept of whether the gastrointestinal tracts of different animals harbor a core microbiota that is shared across the species has been evaluated with the suggestion that there is a core ‘healthy’ microbial population that differs from that of individuals with metabolic disorder(s) (Jalanka-Tuovinen et al., 2011). While an apparent core microbiome in the human gut has been demonstrated in several studies, there is no clear definition of what constitutes a core and, the size of the core population seems to vary between studies (Turnbaugh et al., 2009). We have shown that a phylogenetic core bacterial community exists in all regions of the large intestine of healthy horses (Dougal et al., 2013). However, this study confirmed earlier observations that the core community of the pony is smaller than that found in the rumen of the cow and, unlike core communities that have been identified from other environments; the equine microbiome does not appear to be dominated by any specific OTUs (Dougal et al., 2012, 2013, 2014, 2017).

In addition to phylum and genera-level differences between host-phenotype groups, data from the current study, suggest that for ponies at least, the impacts of body fat content and markers of insulin dysregulation on the composition of the fecal microbiome were greater than those of age. Additionally, aged animals that were also obese clustered more clearly and were more distinctly associated with obese as opposed to aged animals. Importantly, as highlighted by recent murine studies (Xiao et al., 2017), this study provided clear evidence that phenotypic differences in the gut microbiome persist in ponies in the absence of dietary differences.

A relationship between the fecal metabolome (determined both by targeted and non-targeted analysis); the fecal microbiome and host phenotype was not established in the current study. These data contrast with human and murine studies in which host phenotype has been shown to influence both the microbiome and metabolome (Theriot et al., 2014; Zierer et al., 2018). It is likely that differences in digestive anatomy are responsible for this anomaly. Unlike the monogastric condition of mouse and man, in the hind-gut fermenting horse, digesta rapidly pass through the foregut and enter the caecum (52–60 L) within 2–3 h of ingestion. Caecal digesta are retained for 1.5–5 h (dependant on the physical consistency of the diet) to allow initial microbial fermentation. From the caecum, digesta pass into the right ventral colon (retention time of 3 h, 29–32 L) where the largest proportion of fiber fermentation takes place and continues during passage through the remainder of the large intestine. The gastrointestinal tracts of man and mouse are markedly less complex, with significantly more rapid digesta passage through the small and large intestine. Thus, as discussed above, the fecal microbiome, and by implication the fecal metabolome, are not indicative of the complete digestive tract in horse which might help to explain the apparent disconnect between the fecal metabolome and host phenotype. Alternatively, it could be conjectured that the microbiome of the equine hind-gut has the resilience to endure marked alterations in composition with minimal perturbation of fermentation patterns.

The current study has identified that host-phenotype has a major effect on the structure of the microbial population of equine feces. However, clear biomarkers of animal phenotype were not identified in either the fecal microbiome or metabolome. Further studies are needed to confirm whether this is a result of the relatively small number of animals used in this study or a consequence of the functional redundancy within gut microbial population.

## Data Availability Statement

The raw data supporting the conclusions of this manuscript will be made available by the authors, without undue reservation, to any qualified researcher.

## Author Contributions

PM, CA, and AD were involved in study design, conduct of the animal studies, sample collection, data analyses, interpretation, and manuscript presentation. CN, EJ, and HW conducted the microbiome, metabolome, and bioinformatics studies. CN was involved in data interpretation and manuscript preparation. DG-W was involved in most elements of the work including statistical analyses and manuscript preparation. PH and CB were involved in study design, data interpretation, and manuscript preparation.

## Conflict of Interest Statement

Co-authors PH and CB are employed by the funding organization. Co-author AD is employed by CVS Ltd. The remaining authors declare that the research was conducted in the absence of any commercial or financial relationships that could be construed as a potential conflict of interest.

## Supplementary Material

FIGURE S1 Phylogenetic tree depicting clustering of the fecal bacterial microbiome within animal (*n* = 35).

FIGURE S2 Rarefaction curves.

FIGURE S3 Core phyla within the fecal microbiome of the host-phenotypic groups.

TABLE S1 Univariate analysis of associations between insulin/glucose dynamics as measured by CGIT and outset body fat percentage.

TABLE S2 Relative abundance of bacterial phyla between groups. ANOVA analysis was employed to evaluate group differences in the relative abundance of bacterial phyla, and the resulting *p*-value was adjusted for multiple testing using the Benjamini–Hochberg correction.

TABLE S3 Relative abundance of bacterial genera between groups. ANOVA analysis was employed to evaluate group differences in the relative abundance of bacterial genera, and the resulting *p*-value was adjusted for multiple testing using the Benjamini–Hochberg correction.

TABLE S4 Relative abundance of bacterial OTUs significantly different in abundance between Control and Obese groups following Deseq analysis.

TABLE S5 Relative abundance of bacterial OTUs significantly different in abundance between Control and Aged groups following Deseq analysis.

TABLE S6 Relative abundance of bacterial OTUs significantly different in abundance between Obese and Aged groups following Deseq analysis.

TABLE S7 Relative abundance of bacterial OTUs significantly different in abundance between Control and Obese groups across the two years following Deseq analysis.

TABLE S8 Relative abundance of core bacterial OTUs between groups.
