# Supplementary material for: The Equine Gastrointestinal Microbiome: Impacts of Age and Obesity
Source: Front Microbiol. 2018 Dec 7;9:3017. doi: 10.3389/fmicb.2018.03017 (PMC6293011; doi:10.3389/fmicb.2018.03017)

Figure S1: Phylogenetic tree depicting clustering of the faecal bacterial microbiome within animal (n=35)

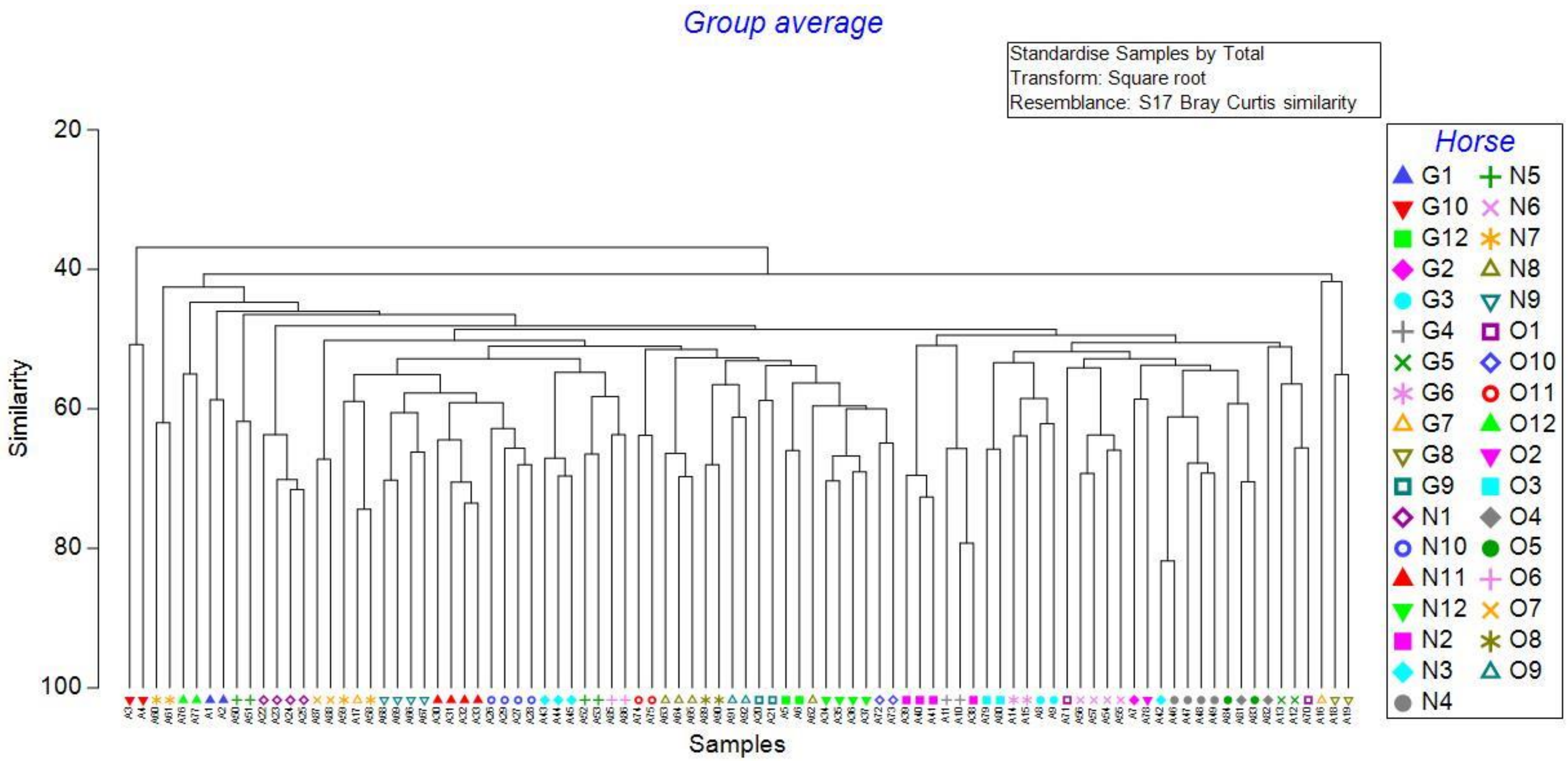

Supplement: FIGURE S1 — Phylogenetic tree depicting clustering of the fecal bacterial microbiome within animal (n = 35). [file Data_Sheet_1.PDF]
