# Supplementary figures and images for: The Equine Gastrointestinal Microbiome: Impacts of Age and Obesity
Source: Front Microbiol. 2018 Dec 7;9:3017. doi: 10.3389/fmicb.2018.03017 (PMC6293011; doi:10.3389/fmicb.2018.03017)

**Figure S2:** Rarefaction curves.

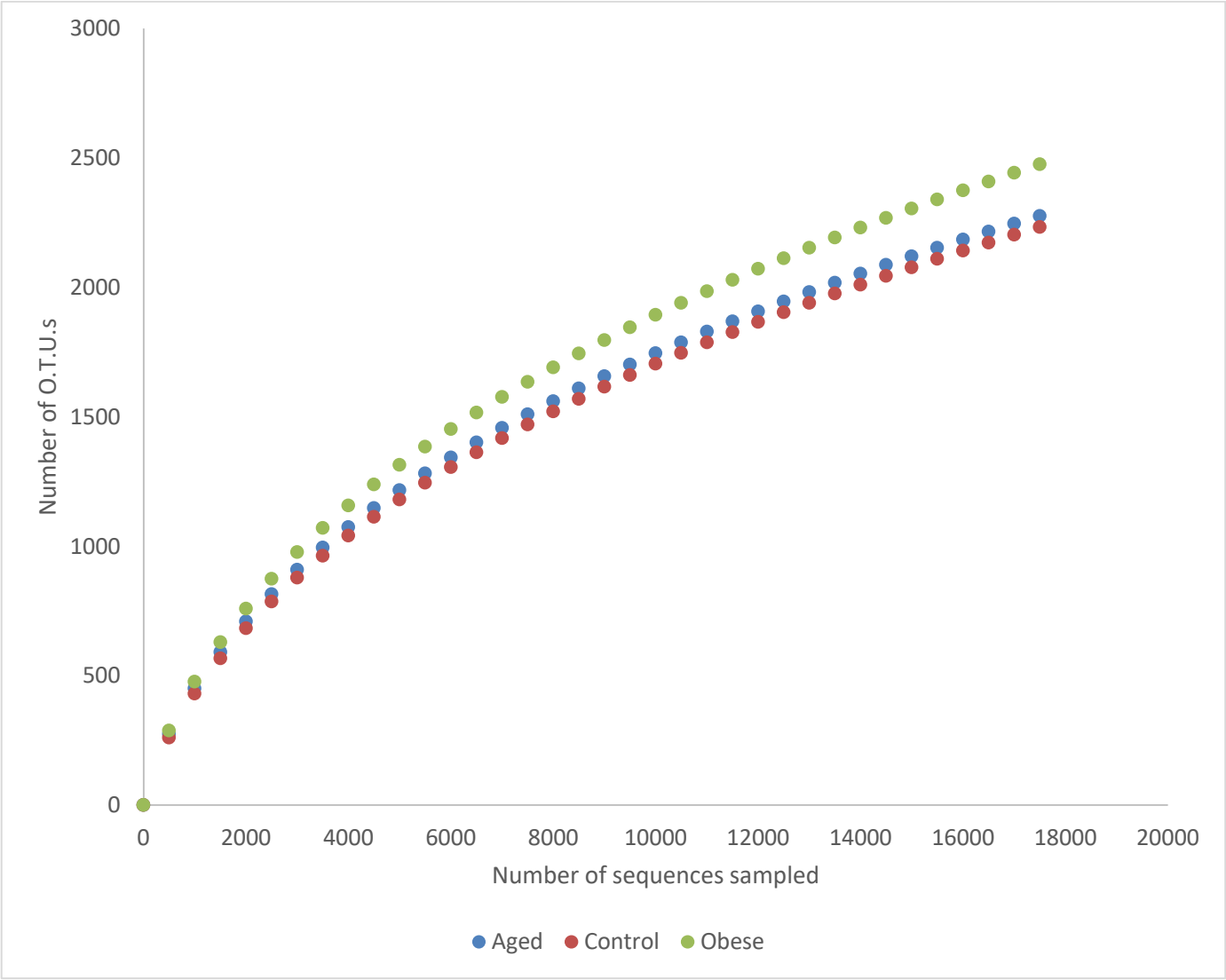

Supplement: FIGURE S2 — Rarefaction curves. [file Data_Sheet_2.PDF]

**Figure S3:** Core phyla within the faecal microbiome of the host-phenotypic groups.

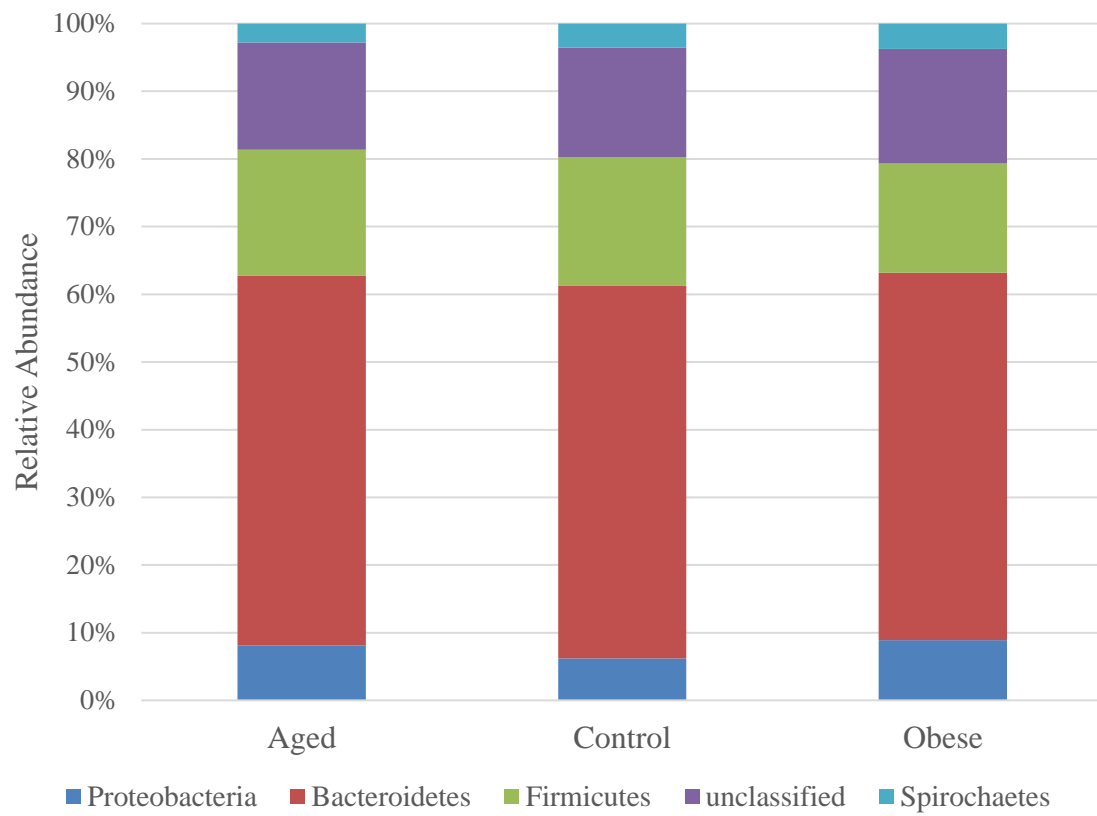

Supplement: FIGURE S3 — Core phyla within the fecal microbiome of the host-phenotypic groups. [file Data_Sheet_3.PDF]
