# Supplementary material for: The Equine Gastrointestinal Microbiome: Impacts of Age and Obesity
Source: Front Microbiol. 2018 Dec 7;9:3017. doi: 10.3389/fmicb.2018.03017 (PMC6293011; doi:10.3389/fmicb.2018.03017)
Supplement: TABLE S1 — Univariate analysis of associations between insulin/glucose dynamics as measured by CGIT and outset body fat percentage. [file Table_1.DOCX]

**Table S1:** Univariate analysis of associations between insulin/glucose dynamics as measured by CGIT and outset body fat percentage.

| **Explanatory variable** | **Coefficient** | **P value** | **95% CI** | **R-squared** |
| --- | --- | --- | --- | --- |
| **Baseline insulin (µIU/ml)** | 0.34 | 0.02 | 0.07 to 0.62 | 0.17 |
| **Baseline** | 0.33 | 0.89 | -4.42 to 5.09 |  |
| **Insulin time 45 (µIU/ml)** | 4.72 | 0.02 | 0.72 to 8.73 | 0.15 |
| **Baseline** | 36.87 | 0.29 | -32.60 to 106.34 |  |
| **Insulin time 75 (µIU/ml)** | 2.49 | 0.12 | -0.68 to 5.67 | 0.07 |
| **Baseline** | 12.73 | 0.64 | -42.33 to 67.79 |  |
| **AUC insulin (µIU/ml/min)** | 222.28 | 0.03 | 23.56 to 421.00 | 0.14 |
| **Baseline** | 1581.23 | 0.36 | -1864.31 to 5026.78 |  |
| **AUC glucose (mmol/L/min)** | 10.01 | 0.01 | 2.58 to 17.44 | 0.19 |
| **Baseline** | 716.85 | < 0.01 | 587.99 to 845.72 |  |
| **Return to baseline glucose concentration (minutes)** | 1.17 | 0.26 | -0.89 to 3.24 | 0.04 |
| **Baseline** | 50.57 | < 0.01 | 14.72 to 86.42 |  |
