# Supplementary material for: The Equine Gastrointestinal Microbiome: Impacts of Age and Obesity
Source: Front Microbiol. 2018 Dec 7;9:3017. doi: 10.3389/fmicb.2018.03017 (PMC6293011; doi:10.3389/fmicb.2018.03017)
Supplement: TABLE S2 — Relative abundance of bacterial phyla between groups. ANOVA analysis was employed to evaluate group differences in the relative abundance of bacterial phyla, and the resulting p-value was adjusted for multiple testing using the Benjamini–Hochberg correction. [file Table_2.DOCX]

**Table S2:** Relative abundance of bacterial phyla between groups. ANOVA analysis was employed to evaluate group differences in the relative abundance of bacterial phyla, and the resulting p-value was adjusted for multiple testing using the Benjamini-Hochberg correction.

|  | **Aged** | **Control** | **Obese** | **SED** | **P-value** | **Benjamini-Hochberg P-value** |
| --- | --- | --- | --- | --- | --- | --- |
| **Fibrobacteres** | 0.108 | 0.187 | 0.100 | 0.037 | <.001 | 0.022 |
| **Bacteroidetes** | 0.434 | 0.406 | 0.451 | 0.013 | 0.004 | 0.022 |
| **Firmicutes** | 0.271 | 0.246 | 0.291 | 0.018 | 0.010 | 0.037 |
| **Actinobacteria** | 0.003 | 0.003 | 0.003 | 0.003 | 0.023 | 0.063 |
| **Proteobacteria** | 0.034 | 0.020 | 0.021 | 0.020 | 0.038 | 0.084 |
| **Synergistetes** | 0.001 | 0.001 | 0.001 | 0.002 | 0.093 | 0.171 |
| **Unclassified** | 0.058 | 0.056 | 0.062 | 0.012 | 0.499 | 0.784 |
| **Tenericutes** | 0.008 | 0.008 | 0.007 | 0.008 | 0.634 | 0.791 |
| **Elusimicrobia** | 0.001 | 0.001 | 0.001 | 0.004 | 0.709 | 0.791 |
| **Candidatus_Saccharibacteria** | 0.001 | 0.001 | 0.001 | 0.003 | 0.719 | 0.791 |
| **Spirochaetes** | 0.045 | 0.044 | 0.046 | 0.010 | 0.830 | 0.809 |
| **SR1** | 0.000 | 0.000 | 0.000 | 0.005 | 0.882 | 0.809 |
