# Supplementary material for: The Equine Gastrointestinal Microbiome: Impacts of Age and Obesity
Source: Front Microbiol. 2018 Dec 7;9:3017. doi: 10.3389/fmicb.2018.03017 (PMC6293011; doi:10.3389/fmicb.2018.03017)
Supplement: TABLE S3 — Relative abundance of bacterial genera between groups. ANOVA analysis was employed to evaluate group differences in the relative abundance of bacterial genera, and the resulting p-value was adjusted for multiple testing using the Benjamini–Hochberg correction. [file Table_3.DOCX]

**Table S3:** Relative abundance of bacterial genera between groups. ANOVA analysis was employed to evaluate group differences in the relative abundance of bacterial genera, and the resulting p-value was adjusted for multiple testing using the Benjamini-Hochberg correction.

|  | **Aged** | **Control** | **Obese** | **SED** | **P-value** | **Benjamini-Hochberg P-value** |
| --- | --- | --- | --- | --- | --- | --- |
| ***Fibrobacter*** | 12.384 | 20.686 | 11.288 | 2.734 | <.001 | 0.017 |
| ***Pseudoflavonifractor*** | 0.291 | 0.120 | 0.373 | 0.066 | <.001 | 0.017 |
| ***unclassified*** | 65.132 | 60.455 | 68.751 | 2.547 | 0.004 | 0.045 |
| ***Anaerovorax*** | 0.249 | 0.207 | 0.274 | 0.025 | 0.017 | 0.131 |
| ***Mogibacterium*** | 0.114 | 0.111 | 0.165 | 0.021 | 0.024 | 0.131 |
| ***Sporobacter*** | 0.206 | 0.207 | 0.259 | 0.020 | 0.024 | 0.131 |
| ***Ruminococcus*** | 0.997 | 0.679 | 0.823 | 0.117 | 0.027 | 0.131 |
| ***Oscillibacter*** | 0.754 | 0.646 | 0.776 | 0.062 | 0.058 | 0.247 |
| ***Prevotella*** | 1.353 | 1.123 | 1.647 | 0.234 | 0.073 | 0.276 |
| ***Phascolarctobacterium*** | 1.584 | 1.226 | 1.579 | 0.196 | 0.083 | 0.282 |
| ***Barnesiella*** | 1.068 | 0.933 | 1.458 | 0.247 | 0.096 | 0.297 |
| ***Pseudomonas*** | 0.536 | 0.002 | 0.004 | 0.276 | 0.133 | 0.353 |
| ***Rhizobium*** | 0.598 | 0.001 | 0.002 | 0.310 | 0.135 | 0.353 |
| ***Faecalitalea*** | 0.242 | 0.178 | 0.258 | 0.051 | 0.198 | 0.468 |
| ***Lachnospiracea_incertae_sedis*** | 0.609 | 0.572 | 0.718 | 0.086 | 0.219 | 0.468 |
| ***Asteroleplasma*** | 0.090 | 0.133 | 0.119 | 0.024 | 0.220 | 0.468 |
| ***Parvibacter*** | 0.120 | 0.100 | 0.126 | 0.019 | 0.302 | 0.525 |
| ***Anaeroplasma*** | 0.663 | 0.540 | 0.504 | 0.096 | 0.314 | 0.525 |
| ***Catabacter*** | 0.202 | 0.087 | 0.091 | 0.078 | 0.315 | 0.525 |
| ***Mobilitalea*** | 0.085 | 0.112 | 0.106 | 0.017 | 0.315 | 0.525 |
| ***Anaerorhabdus*** | 0.698 | 0.609 | 0.330 | 0.227 | 0.324 | 0.525 |
| ***Paraprevotella*** | 1.313 | 1.170 | 1.447 | 0.213 | 0.394 | 0.609 |
| ***Vampirovibrio*** | 0.308 | 0.304 | 0.263 | 0.037 | 0.458 | 0.677 |
| ***Intestinimonas*** | 0.244 | 0.275 | 0.286 | 0.032 | 0.503 | 0.691 |
| ***Lachnobacterium*** | 0.189 | 0.156 | 0.166 | 0.028 | 0.508 | 0.691 |
| ***Phocaeicola*** | 12.384 | 20.686 | 11.288 | 2.734 | <.001 | 0.017 |
| ***Alloprevotella*** | 0.291 | 0.120 | 0.373 | 0.066 | <.001 | 0.017 |
| ***Rikenella*** | 65.132 | 60.455 | 68.751 | 2.547 | 0.004 | 0.045 |
| ***Ureaplasma*** | 0.249 | 0.207 | 0.274 | 0.025 | 0.017 | 0.131 |
| ***Anaerosporobacter*** | 0.114 | 0.111 | 0.165 | 0.021 | 0.024 | 0.131 |
| ***Coprobacter*** | 0.206 | 0.207 | 0.259 | 0.020 | 0.024 | 0.131 |
| ***Saccharibacteria_genera_incertae_sedis*** | 0.997 | 0.679 | 0.823 | 0.117 | 0.027 | 0.131 |
| ***Treponema*** | 0.754 | 0.646 | 0.776 | 0.062 | 0.058 | 0.247 |
| ***Clostridium IV*** | 1.353 | 1.123 | 1.647 | 0.234 | 0.073 | 0.276 |
